# Use of Tobacco

**Published:** 1889-11-30

**Authors:** 


### USE OF TOBACCO.

All newspapers, from the *Times* downwards, confess to a silly season, so we can scarcely expect that medical journals should be exempt. There are certain subjects which crop up with regularity in professional journals. Either someone thinks he has observed something which has not been observed before, but which is probably as old as the hills, or someone asks a question which has been answered at least a hundred times before, the result being equally irritating to anyone maintaining an ordinary acquaintance with past and present medical literature. Thus, we recently observed in a medical journal—we really forget which—a question regarding the relaxant power of tobacco. Now, tobacco solution used formerly to be used as an injection—for the relief of strangulated hernia, for instance—but since the discovery of chloroform, tobacco has fallen into disuse. When our silly season comes round we shall in future sympathise with the *Tocsin*. It appears there is a Manchester Anti-Narcotic League, the secretary of which, we presume, has been irritating the editor of the *Tocsin* by sending quantities of "papers, pamphlets, leaflets, booklets, catechisms, essays, pledge forms, tracts, and general literature on the subject of tobacco." The *Tocsin* says, that as with the consumption of spirituous liquors, so with regard to tobacco, faddists din their distorted views into the ears of a long-suffering public, with the result of exposing their ignorance on the subject. The *Tocsin* objects to the style of propaganda which drags in the Bible with one hand, and the doctor with the other. Doubtless the abuse of tobacco is great, likewise the adulteration, and the *Tocsin* suggests to the members of the Anti-Narcotic League that it would do well to expend their energies in working for the abolition of bad tobacco. Amongst the general diatribe, the *Tocsin* notices a paper headed "Should Women Smoke?" and the *Tocsin* answers the question thus: "Apart from those who could enjoy smoking in a rational way, there are some in whom it might engender a faculty of thought, which is in some conspicuous by its absence, and hence these would be the better able to bring up their children (if any), to look after their husbands (if married), or if not to worship the man divine, according to their orthodox mission in life." The latter it is especially difficult to do without thought. The *Tocsin* further observes that in an ideal existence we should probably be all teetotallers and non-smokers, as we should be so full of health and spirits that no artificial incentives would be required. And the *Tocsin* says, when we get rid of all anti-narcotic leagues, doctors, diseases, most men and many women, we will turn the matter over seriously. If anti-leagues of sorts were generally treated in this spirit, we should hear a great deal less of faddists about tobacco and various other items.

